# Spike in Rhino-Orbital-Cerebral Mucormycosis Cases Presenting to a Tertiary Care Center During the COVID-19 Pandemic

**DOI:** 10.3389/fmed.2021.645270

**Published:** 2021-05-28

**Authors:** Yousef A. Fouad, Tougan Taha Abdelaziz, Anas Askoura, Mohamed Ibrahim Saleh, Mohammad S. Mahmoud, Doaa Maamoun Ashour, Manar Maamoun Ashour

**Affiliations:** ^1^Department of Ophthalmology, Ain Shams University Hospitals, Cairo, Egypt; ^2^Department of Radiology, Ain Shams University Hospitals, Cairo, Egypt; ^3^Department of Otorhinolaryngology, Ain Shams University Hospitals, Cairo, Egypt

**Keywords:** mucormycosis, COVID-19, SARS-CoV-2, rhino-orbital-cerebral mucormycosis, invasive fungal infection

## Abstract

**Objective:** To determine if there was an increase in the rate of cases presenting with rhino-orbital-cerebral mucormycosis (ROCM) to a tertiary care center during the first wave of the coronavirus disease 2019 (COVID-19) pandemic and the characteristics of the presenting cases.

**Methods:** Retrospective observational study reviewing ROCM cases presenting from March 25 until September 25, 2020. Cases fulfilling the clinical, radiological, and pathological/microbiological criteria for diagnosis with ROCM were included. The number of cases presenting during the designated interval, their COVID-19 status, comorbidities, and clinical presentation were analyzed. The number of cases during the corresponding interval in the previous 3 years was used as reference to detect if there was a recent spike.

**Results:** Of the 12 ROCM cases identified, 5 had a concurrent positive reverse transcription PCR (RT-PCR) test result for severe acute respiratory syndrome coronavirus 2 (SARS*-*CoV*-*2), 1 had a prior positive result, and 6 did not have concurrent nor prior positive test results. Nine of the 12 cases had poorly controlled diabetes mellitus, and 2 cases had a hematological malignancy. All cases had orbital invasion, and eight cases had cerebral invasion. The number of cases identified during the interval is much higher than the numbers presenting in the prior 3 years during equivalent intervals (range, one to two cases) than those reported in the literature in different settings in the pre-pandemic era.

**Conclusions:** There is an increased rate of ROCM cases presenting to our center during the first wave of the COVID-19 pandemic. This is a preliminary report, and further studies are needed to corroborate the findings and explain possible underlying links.

## Introduction

Rhino-orbital-cerebral mucormycosis (ROCM) is a rare life-threatening invasive fungal infection that often occurs in immunocompromised individuals, with around 70% of the cases complicating a diabetic ketoacidosis (DKA) event (1, 2). The condition originates in the nose and paranasal sinuses but is often suspected following orbital spread, explaining its poor prognosis (3). The infiltrating fungus destroys the surrounding bone and soft tissue through vascular thrombosis and subsequent tissue infarction and may reach the brain with fatal complications (2).

The incidence of mucormycosis is often underestimated (4). This could be attributed to the rarity of the condition, declining autopsy rates, occasional unavailability of confirmatory tissue biopsy for diagnosis, and scarcity of population-based studies (4). Furthermore, most epidemiological studies report the incidence of all forms of mucormycosis combined (0.43–1.7 cases per million population) with no subgrouping specific for ROCM incidence (4, 5). Nevertheless, emerging reports (6, 7) have described a rising trend in mucormycosis in the latest decades, which is hypothesized to reflect the growing populations with diabetes mellitus (DM), hematological malignancy, and bone marrow transplants.

Reports on ocular manifestations in patients with the novel coronavirus disease 2019 (COVID-19) describe self-limiting conjunctivitis (8) and rare neuro-ophthalmic manifestations that include optic neuritis and ocular motor cranial neuropathies (9). We are still learning about the overreaching health implications and long-term sequalae associated with the viral pandemic. Separate case reports (10–14) have been emerging describing ROCM coinfection in COVID-19-positive patients. We have observed an increased rate of ROCM cases in our hospitals during the first wave of the pandemic. We set out to identify if this were a true increase and, should it be, review the COVID-19 status of the patients, other associated comorbidities, and clinical manifestations.

## Methods

We retrospectively reviewed cases presenting with ROCM to the tertiary care of Ain Shams University Hospitals over the 6 months from March 25, 2020 (when the government imposed partial lockdown due to spike in COVID-19) to September 25, 2020. Case identification was based on the global guideline for the diagnosis and management of mucormycosis (15), which includes patients presenting with (1) symptoms of sinusitis, facial pain or swelling, proptosis, or decreased vision; (2) radiological evidence of bone destruction and orbit/brain infiltration; and (3) tissue biopsy with pathological evidence of tissue invasion, vessel occlusion, and non-pigmented non-/pauci-septate hyphae with variable width (6–16 μm) or the same typical fungal appearance in microbiological culture.

For all cases, the reverse transcription PCR (RT-PCR) result for severe acute respiratory syndrome coronavirus 2 (SARS-CoV-2), which was routinely obtained on admission, the history, the clinical presentation and course, and the radiological findings were recorded. We also reviewed our records for ROCM cases presenting during the same months of the three preceding years (2017–2019).

The work was approved by the ethical committee of the faculty of medicine, Ain Shams University, with written informed consents obtained from patients (or next of kin in case of patient's death).

## Results

Twelve cases with ROCM were identified over 6 months (compared to one case in 2017, two in 2018, and one in 2019, all in the corresponding 6-month interval), six of which were male. The demographic and clinical data of the cases are detailed in Table 1. The mean (SD) age of the cases was 51.2 (16.7) years. Five of the 12 cases (41.7%) had a positive SARS-CoV-2 RT-PCR test result at the time of diagnosis with ROCM. One case (8.3%) tested negative for COVID-19 at the time of diagnosis but had a prior positive test record and had been admitted for treatment, with symptoms of ROCM appearing 2 days following discharge. The remaining six cases (50%) had negative SARS-CoV-2 RT-PCR results on diagnosis of ROCM and no prior record of positive COVID-19 testing. All patients concurrently positive for SARS-CoV-2 had moderate to severe disease and were treated for COVID-19 in accordance with the Egyptian national guidelines for COVID-19 (16) that included systemic glucocorticoids in severe cases (three patients) and hydroxychloroquine in all patients.

**Table 1:** Demographic and clinical data of the 12 cases presenting with rhino-orbital-cerebral mucormycosis (ROCM).

**No**.	**Age**	**Sex**	**Comorbidities**	Diabetic control^*^	COVID testing^**^	**Cerebral invasion**	**Debridement**	**Outcome**
1	63	F	DM	Poor	Prior	√	√	Recovery
2	49	F	DM, CKD	Poor	Negative	√		Death
3	69	F	DM, IHD	Poor	Negative	√	√	Death
4	55	M	DM	Poor	Positive	√		Death
5	54	M	DM, CKD, IHD	Good	Positive			Death
6	67	M	DM, CKD	DKA	Positive			Death
7	41	F	DM	Poor	Positive	√	√	Recovery
8	42	M	DM	Poor	Positive		√	Recovery
9	16	M	ALL	NA	Negative		√	Recovery
10	28	F	DM	Poor	Negative	√	√	Recovery
11	65	M	ALL, HTN	NA	Negative	√		Death
12	65	F	DM	Poor	Negative	√	√	Recovery

* *On presentation (control status based on HbA1C level, poor if >8%, and good if ≤8%)*.

** *RT-PCR for SARS-CoV-2, results on admission or prior documentation of a positive result*.

*DM, diabetes mellitus; CKD, chronic kidney disease; IHD, ischemic heart disease; ALL, acute lymphoblastic leukemia; HTN, hypertension; DKA, diabetic ketoacidosis; NA, not applicable*.

Ten of the 12 cases (83.3%) had DM that was poorly controlled in 9 of them at the time of occurrence of ROCM, with a median HbA1c of 9.7%. One of the 10 cases with DM had been previously undiagnosed and had his first presentation of DKA with coexisting ROCM. The two remaining non-diabetic cases (16.7%) had a known underlying hematological malignancy (acute lymphocytic leukemia) and had received recent chemotherapy. Three cases (25%) had chronic kidney disease, and one case (8.3%) had ischemic heart disease.

**Figure 1:**
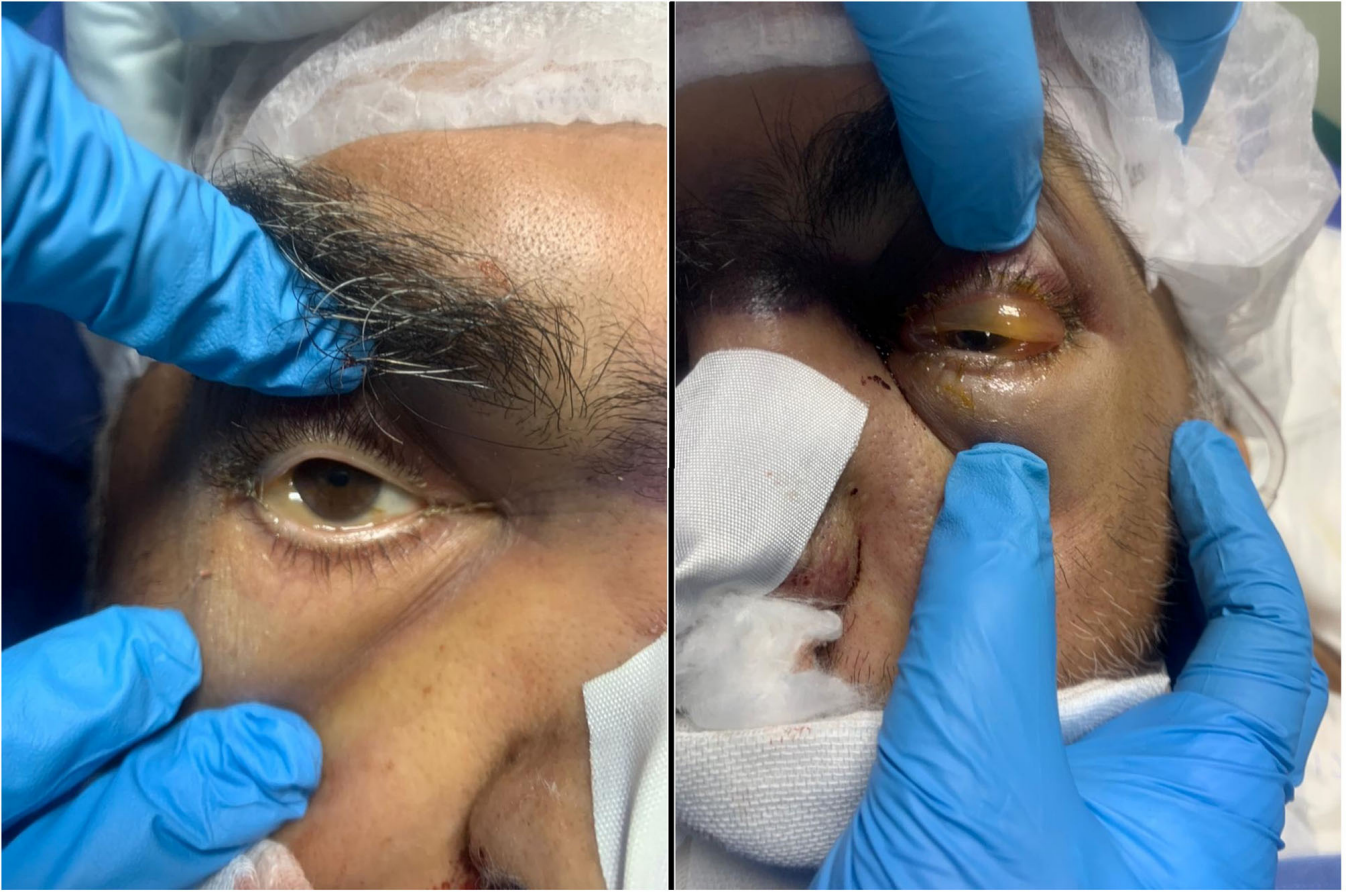
Presenting signs in one of the cases. Moderate conjunctival chemosis in the right eye and severe conjunctival chemosis with lid swelling in the left eye in a 54-year-old male patient, diabetic, with chronic kidney disease, on mechanical ventilation in the intensive care unit for severe COVID-19.

**Figure 2:**
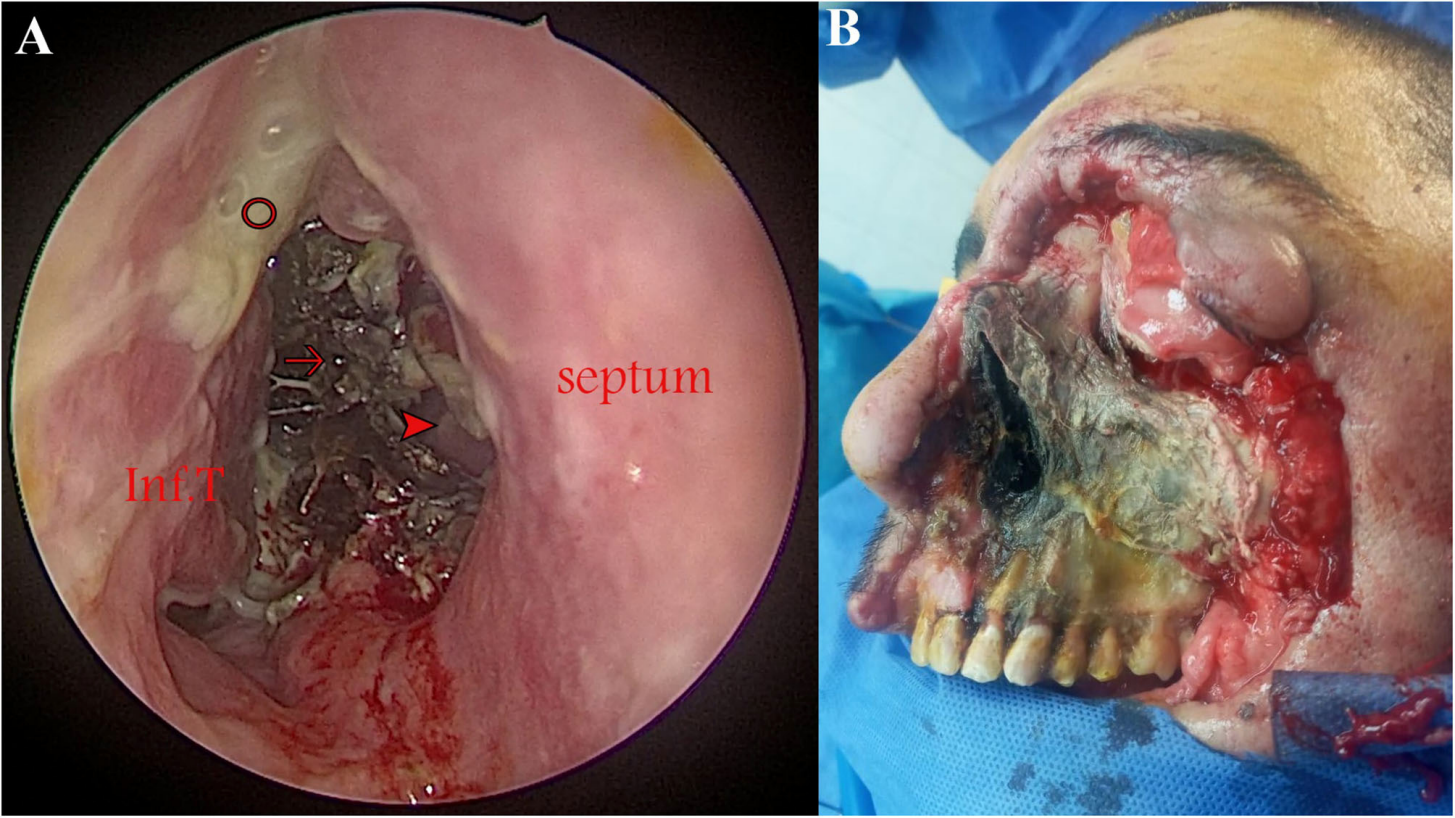
Advanced rhino-orbital-cerebral mucormycosis (ROCM) in two patients. **(A)** Endoscopic view (0° lens) demonstrating black necrotic tissue filling the right nasal cavity (arrow), mucopurulent secretions (circle), and a perforation in the bony septum (arrowhead) through which the left inferior turbinate (Inf.T) can be seen. **(B)** Progressed disease despite surgical debridement in a 55-year-old diabetic male patient who had tested positive for COVID-19.

**Figure 3:**
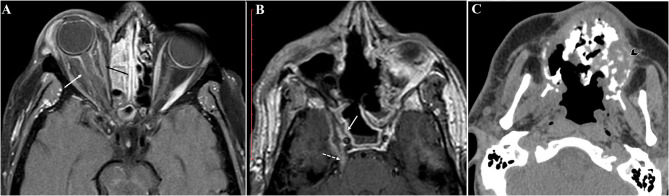
Radiological findings in our rhino-orbital-cerebral mucormycosis (ROCM) cases. Axial postcontrast T1WI showing **(A)** orbital infiltration with optic nerve sheath inflammatory changes (white arrow), the extra ocular muscles are hypoenhancing (black arrow) secondary to ischemic changes, and **(B)** cavernous sinus infiltration with lack of expected enhancement (solid white arrow). Perineural spread of the fungus is seen along the trigeminal nerve (dashed white arrow). **(C)** Axial non-contrast CT reveals destructive sinonasal soft tissue mass with fragmented bone and air loculi secondary to osteonecrosis (arrowhead).

The initial presenting signs of the cases were lid edema (50%), conjunctival chemosis (50%, Figure 1), diminution of vision (41.7%), proptosis (33.3%), facial edema (25%), nasal crusts (25%, Figure 2A), total ophthalmoplegia (16.7%), and paralytic esotropia (8.3%). On radiological assessment (Figure 3), all cases developed orbital infiltration with two cases (16.7%) having bilateral involvement. Four cases (33.3%) developed cavernous sinus infiltration, two cases (16.7%) developed internal carotid artery infiltration, and two cases (16.7%) developed a cerebral abscess. COVID-19 coinfection was neither associated with specific presenting signs of ROCM nor did it affect our management course. All cases received medical treatment for ROCM including measures to control the general condition, underlying risk factors, and complications, together with systemic antifungals and antibiotics to prevent secondary bacterial infection. Seven cases (58.3%) were fit to undergo surgical debridement (Figure 2B), five of which (71.4%) had a non-salvageable eye globe on the affected side that was sacrificed during surgery. The final outcome was recovery and discharge in seven patients (58.3%) and death due to complication in five patients (41.7%), three of which had COVID-19 and two had received systemic steroids.

## Discussion

We report a higher rate of ROCM cases presenting to our care during the COVID-19 pandemic in comparison to the same period in the previous 3 years. Half of the cases had a positive test record of SARS-CoV-2 infection. We cannot exclude a prior undocumented, untreated, or asymptomatic infection with SARS-CoV-2 in the other half. Multiple case reports and series (10–14) from different parts of the world have described ROCM—or invasive maxillofacial fungal infection—in SARS-CoV-2-positive patients but with no evidence of an actual spike in case numbers.

Possible implication of COVID-19 in the development of ROCM may include impaired host defenses against the fungus by viral-induced lymphopenia or the therapeutic use of corticosteroids and/or hydroxychloroquine, both likely to impair phagocytic immune-cell response, which is the major defense mechanism against mucormycosis (2). It is worth noting that the Egyptian national guidelines for COVID-19 (16) during the examined interval included hydroxychloroquine treatment for all diagnosed cases regardless of severity and hydrocortisone for severe cases. Other implicated factors for COVID-19 in ROCM development may include aggravated disruption of blood sugar control and general debilitation in diabetic patients and/or late seeking of medical care during lockdown resulting in more DKA events.

There further remains the remote possibility that our reported spike is an incidental one, partly attributable to increased referral of complicated cases to our more-equipped center, especially since we report the experience of a single center due to the lack of a national registry. However, 12 ROCM cases presenting over the period of 6 months to a single center is still a high rate when viewed in the context of literature reports. For example, in a previous report (17) about ROCM from the University of California, Los Angeles, 28 cases were diagnosed in all centers over a period of 40 years. Another report (3) from North Carolina described 13 cases of ROCM presenting over a period of 14 years. A more recent report (18) that included ROCM cases from two large medical institutes in Mumbai, India encompassed 20 cases over the period of 3 years.

Future work should attempt to elucidate whether a causal link exists between COVID-19 and ROCM. We, thus, call on practitioners who noticed any recent spike in rates of invasive fungal sinusitis to report their experience. Until we have more answers, we stress on adopting early clinical suspicion when encountering cases with similar presentation.

## Data Availability Statement

The raw data supporting the conclusions of this article will be made available by the authors, without undue reservation.

## Ethics Statement

The studies involving human participants were reviewed and approved by Research Ethics Committee, Faculty of Medicine, Ain Shams University. The patients/participants provided their written informed consent to participate in this study. Written informed consent was obtained from the individual(s) for the publication of any potentially identifiable images or data included in this article.

## Author Contributions

YF, TA, and DA shared in writing the manuscript. AA, MS, MM, and MA shared in reviewing and editing the final version of the work. All authors shared in the conception of the idea of the manuscript, data curation, and interpretation.

## Conflict of Interest

The authors declare that the research was conducted in the absence of any commercial or financial relationships that could be construed as a potential conflict of interest.
